# Prenatal care associated with neonatal outcomes in maternity hospitals: a hospital-based cross-sectional study*

**DOI:** 10.1590/1980-220X-REEUSP-2023-0145en

**Published:** 2024-02-09

**Authors:** Eglídia Carla Figueirêdo Vidal, Lara Leite de Oliveira, Camila Almeida Neves de Oliveira, Marianne Maia Dutra Balsells, Maria Aline Rodrigues Barros, Emery Ciana Figueirêdo Vidal, Ana Karina Bezerra Pinheiro, Priscila de Souza Aquino

**Affiliations:** 1 Universidade Regional do Cariri, Centro de Ciências Biológicas e da Saúde, Departamento de Enfermagem, Crato, CE, Brazil.; 2 Universidade Federal de Sergipe, Centro de Ciências Biológicas e da Saúde, Departamento de Enfermagem, Aracaju, SE, Brazil.; 3 Universidade Regional do Cariri, Centro de Ciências Biológicas e da Saúde, Colegiado de Enfermagem, Iguatu, CE, Brazil.; 4 Universidade Federal do Ceará, Faculdade de Farmácia, Odontologia e Enfermagem, Departamento de Enfermagem, Fortaleza, CE, Brazil.

**Keywords:** Prenatal Care, Maternal and Child Health, Nursing, Obstetrics, Maternal Health Services, Atención Prenatal, Salud Materno-Infantil, Enfermería, Obstetricia, Servicios de Salud Materna, Cuidado Pré-Natal, Saúde Materno-infantil, Enfermagem, Obstetrícia, Serviços de Saúde Materna

## Abstract

**Objective:**

To verify the association between prenatal care quality indicators and neonatal outcomes in maternity hospitals.

**Method:**

Hospital-based cross-sectional study in four high-risk referral maternity hospitals in the five health macro-regions enabled by the Stork Network in Ceará-Brazil. Between April 2017 and July 2018, 440 puerperal women were interviewed using simple probabilistic sampling and a formula with finite populations and stratification of each maternity hospital. The analysis involved Pearson's Chi-Square, Adjusted Residuals Analysis and Fisher's Exact.

**Results:**

There was an association between fewer consultations with prematurity and low birth weight. Delivery in the maternity hospital where the woman lived was associated with low birth weight and the need for ventilatory support.

**Conclusion:**

Prenatal care quality indicators influenced neonatal outcomes, which underlines the importance of ensuring access and quality of care as ways of reducing infant morbidity and mortality.

## RESUMO

**Objetivo:**

Verificar a associação entre os indicadores da qualidade do pré-natal e os desfechos neonatais em maternidades.

**Método:**

Estudo transversal de base hospitalar, em quatro maternidades referências para alto risco nas cinco macrorregiões de saúde habilitadas na Rede Cegonha no Ceará-Brasil. Realizou-se entre abril de 2017 e julho de 2018, entrevista com 440 puérperas, por amostragem probabilística simples e fórmula com populações finitas e estratificação de cada maternidade. A análise envolveu o Qui-Quadrado de Pearson, Análise de Resíduos Ajustados e Exato de Fisher.

**Resultados:**

Nota-se associação entre menor número de consultas com prematuridade e baixo peso ao nascer. Parto na maternidade de residência da mulher esteve associada com baixo peso ao nascer e necessidade de suporte ventilatório.

**Conclusão:**

Indicadores de qualidade do pré-natal influenciaram os desfechos neonatais, o que afirma a importância da garantia de acesso e qualidade da assistência como formas de reduzir a morbimortalidade infantil.

## RESUMEN

**Objetivo:**

Verificar la asociación entre los indicadores de calidad de la atención prenatal y los resultados neonatales en las maternidades.

**Método:**

Estudio transversal de base hospitalaria en cuatro maternidades de referencia de alto riesgo en las cinco macrorregiones sanitarias autorizadas por la Red Cigüeña en Ceará-Brasil. Entre abril de 2017 y julio de 2018, se entrevistaron 440 puérperas mediante muestreo probabilístico simple y fórmula con poblaciones finitas y estratificación de cada maternidad. El análisis involucró Chi-Cuadrado de Pearson, Análisis de Residuos Ajustados y Exacto de Fisher.

**Resultados:**

Hubo asociación entre menor número de consultas con prematuridad y bajo peso al nacer. El parto en el hospital de maternidad donde vivía la mujer se asoció con el bajo peso al nacer y la necesidad de asistencia ventilatoria.

**Conclusión:**

Los indicadores de calidad de la atención prenatal influyeron en los resultados neonatales, lo que subraya la importancia de garantizar el acceso y la calidad de la atención como formas de reducir la morbimortalidad infantil.

## INTRODUCTION

Prenatal care (PN) encompasses the systematic monitoring of pregnant women from the beginning of pregnancy, with the aim of achieving the best maternal and neonatal outcomes^(1)^. Quality prenatal care is essential for health, as it involves health promotion actions for the maternal-fetal binomial, identifying possible unfavorable situations early on and enabling timely interventions^(2–4)^.

As the main source of prenatal care in Brazil, the Unified Health System (SUS) still has shortcomings in the continuity and quality of care provided^(5)^. The specialized national literature^(1)^ also points out that the challenges persist with a high level of inadequacies in PN care in Brazil. These inadequacies in prenatal care are associated with negative outcomes, ranging from prematurity and low birth weight (LBW) to a high risk of maternal and fetal death, admissions to intensive care units, among others^(2)^.

In this sense, when it came to presenting quality indicators for prenatal care, it was shown that only 15% of women received adequate prenatal care and the situation was more serious among younger women, those with lower family incomes and those living in the North and Center-West regions^(3)^. A recent study^(6)^ evaluating the adequacy of prenatal care found widespread coverage in Brazil, but inequality and poor quality of care, especially among women in the country’s poorest regions.

From this perspective, it can be inferred that early neonatal mortality presupposes causes of death related to the precarious care offered to women during pregnancy and childbirth, as well as inadequate care in delivery rooms and neonatal units^(7)^. Therefore, a set of actions is needed to reduce infant mortality rates, by improving the provision of services from the time of birth onwards, supported by continuous health surveillance and the promotion of healthier lifestyles so that negative maternal and neonatal outcomes can be avoided.

The analysis of quality indicators for prenatal care and childbirth, such as number of consultations, gestational age at onset of prenatal care, immunization, examinations, professional who carried out the prenatal care, risk stratification and place of delivery, as well as their association with neonatal outcomes, is important for identifying gaps in care that require intervention.

However, recent studies^(1)^ have shown gaps that have a significant impact on perinatal outcomes, ranging from the late start of prenatal care to pregnant women’s pilgrimage in search of a referral maternity hospital. The scarcity of studies addressing this issue at a state level is therefore noteworthy, and this is the first study carried out in Ceará involving maternity hospitals that are benchmarks for good practices in normal childbirth care, according to the Stork Network Strategy^1^.

In fact, this thematic network of care is presented as a network of care aimed at improving maternal and child care, through an organizational arrangement of the Unified Health System (SUS), structured with the aim of ensuring women’s right to reproductive planning and humanized care during pregnancy, childbirth and the puerperium; as well as for children, the right to safe birth and healthy growth and development^(8)^.

In view of the above, the aim was to verify the association between prenatal care quality indicators and neonatal outcomes in maternity hospitals in Ceará.

## METHOD

### Study Design

This is a cross-sectional study. The study report followed the Strengthening the Reporting of Observational Studies in Epidemiology (STROBE) guidelines^(9)^.

### Study Site

The study was carried out in four maternity hospitals, which are referral centers for high-risk pregnancies (HRP) in obstetric care for the five health macro-regions (MRs), qualified under the Stork Network (SN) in Ceará, from April 2017 to July 2018. The MRs were Fortaleza, Sobral, Sertão-Central and Cariri. The East Coast macro-region, at the time of this study, had this type of reference directed to the Fortaleza MR.

It should be noted that the Stork Network, established in 2011, is a strategy of the Brazilian federal government that proposes improving care for women during pregnancy, childbirth and the postpartum period, as well as for newborns and children up to two years of age, by coordinating the different points of health care, the logistics system, the support system and the governance of the health care network^(8)^.

### Population and Selection Criteria

The population was composed of puerperal women. The inclusion criteria were: being admitted to the maternity hospitals of reference at least 12 hours postpartum. The following exclusion criteria were defined: puerperal women who had communication limitations, complications in their clinical condition that prevented them from answering the survey form and birth outcome with a stillborn child.

### Sample Definition

The sample of puerperal women was a simple probability sample, considering a 95% confidence interval, a significance level of 0.05, a maximum error of 5% and a prevalence of 50%. A sample calculation was carried out using a formula for studies with finite populations, based on deliveries in the year prior to collection (n = 6042), with the following representative stratification of deliveries in each maternity hospital of reference in the MR: 39% in the MR Fortaleza, 29% in the MR Sobral, 20% in the MR Sertão Central and 12% in the MR Cariri. A rate of 10% was added for possible loss of data during collection, culminating in the final sample of 440 puerperal women admitted to the MR reference maternity hospitals in Ceará, respectively 179 MR Fortaleza, 134 MR Sobral, 77 MR Sertão-Central and 50 MR Cariri.

### Data Collection

Data was collected between April 2017 and July 2018, by means of an interview on the premises of the maternity hospitals, specifically in the rooming-in area. The companion was asked not to be present at the time. The average interview lasted 40 minutes.

For data collection, two forms were used which were validated in terms of appearance and content by seven expert judges using the Delphi technique, whose face and content validation process lasted eight hours in face-to-face meetings. The criteria for the experts involved teaching and practical experience in Nursing courses and Women’s Health or similar subjects, for at least five years and publications of articles in the thematic area of women’s health. In addition, data from medical records and the PN card were also used.

The predictor variables were established considering the quality of prenatal care and childbirth: early onset of prenatal care (up to 12 weeks); adequate number of appointments (greater than or equal to 7); immunization during pregnancy (influenza, DtaP and hepatitis B); HIV and syphilis serology; professional who performed the prenatal care; complications during pregnancy; risk stratification on admission to the maternity hospital and childbirth in the maternity hospital in the municipality of residence. The cutoff point was the start of prenatal care at 12 gestational weeks and at least seven consultations, as recommended by the Ministry of Health (8). As for neonatal outcomes, the following were assessed: Spontaneous prematurity – classified as a gestational age of less than 37 gestational weeks, in which the onset of labor was spontaneous or with premature rupture of the membranes; Low birth weight (<2500g), Apgar score at the 1st and 5th minute (<7); Ventilatory support for the NB after birth; and, Immediate neonatal resuscitation at birth, taking other evaluative surveys of this nature as a parameter^(5)^.

### Data Analysis and Processing

The data was analyzed using the Statistical Package for the Social Sciences® (SPSS), version 24.0. The statistical tests used to analyze the associations between the independent variables (obstetric and care) and the neonatal outcomes involved Pearson’s Chi-square test, complemented by the Adjusted Residuals Analysis and Fisher’s Exact test. A value of p < 0.05 was used to consider statistical significance.

### Ethical Aspects

In compliance with Resolution 466/12 of the National Health Council/Ministry of Health^(10)^, the ethical issues were cleared by the Ethics Committees (Opinions No. 1.939.946/MEAC and No. 2.531.361/UNICATÓLICA), with the due consent of each institution. All participants signed the Free and Informed Consent Form (FICF) in two copies.

## RESULTS

The sociodemographic characterization of the 440 puerperal women showed that 81.3% (n = 357) were over 19 years old. As for schooling, 57.6% (n = 250) had more than 10 years of schooling; 61.1% (n = 254) had a monthly family income of less than or equal to one Brazilian minimum wage (i.e. US$298.00 per month in 2017), and 51.5% (n = 217) had no paid occupation, revealing the prevalent context of poverty in the sample. As for race/skin color, 81% (n = 356.4) declared themselves to be brown or black and 80.9% (n = 356) of the women lived with a partner.

With regard to obstetric characteristics, 57.7% (n = 254) had a previous pregnancy or more, and of these, 82.9% (n = 68) reported a history of miscarriage; 50.6% of the women (n = 222) were primiparous and, of the 49.4% (n = 217) of secundiparous and multiparous women, 95.0% (n = 207) had previously given birth vaginally. Table 1 below shows the association between PN care indicators and neonatal outcomes.

**Table 1:** Association of PN care indicators with maternity hospitals by health macro-region enabled by the Stork Network – Fortaleza, CE, Brazil, 2018.

Variables for PN care	Macro region					p-value*
	Cariri n (%)	Fortaleza n (%)	Sertão central n (%)	Sobral n (%)	Ceará n (%)	
**Start of PN**						
Up to 12 weeks	40 (80%)^(+)^	90 (50.3%)^(⁻)^	46 (59.7%)	80 (59.7%)	256 (58.2%)	**0.002**
Over 12 weeks	10 (20%)^(⁻)^	89 (49.7%)^(+)^	31 (40.3%)	54 (40.3%)	184 (41.8%)	
**Number of PN visits**						
Up to 6	10 (20.4%)^(⁻)^	77 (52.7%)^(+)^	24 (31.2%)	35 (27.5%)	146 (34.1%)	**0.002**
7 or more	39 (79.6%)^(+)^	98 (34.8%)^(⁻)^	53 (68.8%)	92 (72.4%)	282 (65.9%)	
**Vaccines during pregnancy**						
Yes	50 (100%)	168 (96%)	68 (90.7%)^(⁻)^	130 (99.2%)^(+)^	416 (96.5%)	**0.006**
No	0 (0%)	7 (4%)	7 (9.3%)^(+)^	1 (0.8%)^(⁻)^	15 (3.5%)	
**HIV tests during pregnancy**						
Yes	48(96%)	157(90.2%)	65(87.8%)	119(90.8%)	389(90.7%)	0.489
No	2(4%)	17(9.8%)	9(12.2%)	12(9.2%)	40(9.3%)	
**Tests for syphilis in pregnancy**						
Yes	47(94%)	154(86.5%)	70(90.9%)	104(78.8%)^(⁻)^	375(85.8%)	**0.020**
No	3(6%)	24(13.5%)	7(9.1%)	28(21.2%)^(+)^	62(14.2%)	
**Professional who carried out the PN**						
Both	41(83.7%)	128(74.4%)^(⁻)^	62(83.8%)	113(86.3%)	344(80.7%)	**0.004**
Nurse	3(6.1%)	15(8.7%)	6(8.1%)	15(11.5%)	39(9.1%)	
Physician	5(10.2%)	29(16.9%)^(+)^	6(8.1%)	3(2.3%)^(⁻)^	43(10.2%)	
**Complications during pregnancy**						
Yes	9 (18%)	36 (49.7%)^(+)^	18 (24.7%)^(+)^	4 (3.1%)^(⁻)^	67 (15.5%)	**<0.001**
No	41 (82%)	142 (79.8%)^(⁻)^	55 (75.3%)^(⁻)^	127 (96.9%)^(+)^	365 (84.5%)	
**Risk stratification at admission**						
High risk	6 (12%)	27 (15.1%)	15 (19.5%)	17 (12.7%)	65(14.7%)	0.544
Normal risk	44 (88%)	152 (84.9%)	62 (80.5%)	117 (87.3%)	375(85.3%)	
**Delivery at municipality of residence**						
Yes	21(42%)	133(74.3%)^(+)^	33(43.4%)	44(32.8%)^(⁻)^	231(52.6%)	**<0.001**
No	29(58%)	46(25.7%)^(⁻)^	43(56.6%)	90(67.2%)^(+)^	208(47.4%)	
**Prematurity (<37 weeks)**						
Yes	9(18%)	35(21.1%)	7(9.9%)^(⁻)^	27(21.3%)	78(18.8%)	0.187
No	41(82%)	131(78.9%)	64(90.1%)^(+)^	100(78.7%)	336(81.2%)	
**Low birth weight (<2500g)**						
Yes	4(8%)	24(14.5%)	9(11.7%)	25(18.8%)	62(14.1%)	0.222
No	46(92%)	155(85.5%)	68(88.3%)	108(81.2%)	377(85.9%)	
**1st minute Apgar**						
Up to 7	5(11.4%)	28(15.7%)	3(4%)^(⁻)^	23(17.6%)	59(13.8%)	**0.039**
Over 7	39(88.6%)	150(84.3%)	72(96%)^(+)^	108(82.4%)	369(86.2%)	
**5th minute Apgar**						
Up to 7	3(6.8%)^(+)^	2(1.1%)	1(1.4%)	3(2.3%)	9(2.1%)	0.122
Over 7	41(93.2%)^(⁻)^	176(98.9%)	73(98.6%)	129(97.7%)	419(97.9%)	
**Ventilatory support for NB after birth**						
Yes	5(10%)	16(8.9%)^(⁻)^	7(9.1%)	29(21.6%)^(+)^	57(12.9%)	**0.005**
No	45(90%)	163(91.1%)^(+)^	70(90.9%)	105(78.4%)^(⁻)^	383(87.1%)	
**Immediate neonatal resuscitation**						
Yes	1(2%)	3(1.7%)	0(0%)	4(3%)	8(1.8%)	0.479
No	49(98%)	176(98.3%)	77(100%)	130(97%)	432(98.2%)	

*Pearson Chi Square; ^(+)^ Significant positive association; ^(⁻)^ Significant negative association.

In fact, prenatal care showed regional variations in the indicators for the start of prenatal care, namely: early start of prenatal care, complications during pregnancy, number of consultations, immunization during pregnancy, tests for syphilis during pregnancy, professional who carried out the prenatal care, delivery in the maternity hospital in the municipality of reference, 1st minute Apgar score and ventilatory support for the NB after birth.

As can be seen in Table 1, some statistical associations were found. The Cariri MR had a higher-than-expected prevalence of early PN onset, to the detriment of the higher prevalence of late PN onset in the Fortaleza MR. The Fortaleza MR stood out for its discrepant prevalence of up to 6 consultations, to the detriment of the Cariri region, with a higher prevalence of 7 or more consultations. The Sobral MR had a higher-than-expected prevalence of immunization during pregnancy, in comparison with the Sertão Central MR.

Regarding the tests to detect syphilis during pregnancy, the Sobral MR stood out with the lowest prevalence. When it came to the professional who carried out the prenatal care, the Fortaleza MR had a higher prevalence of doctors compared to the Sobral MR, which had a lower prevalence. In addition, the Fortaleza and Sertão Central MRs had a higher-than-expected prevalence of pregnancy complications than the Sobral MR. The Fortaleza MR had a higher prevalence of births in the municipality of residence, while the Sobral MR had a lower prevalence of births in the municipality of Sobral.

In fact, the associations between neonatal outcomes and the MRs showed that the Apgar score in the first minute up to 7 was less prevalent in the MR Sertão Central and that there was a greater need for ventilatory support after delivery in the MR Sobral, to the detriment of the MR Fortaleza, which had a lower prevalence than expected.

Table 2 shows the association between the PN care variables and the neonatal outcomes observed in reference maternity hospitals for high-risk pregnancies in the health macro regions of Ceará.

**Table 2:** Association between PN care indicators and neonatal outcomes – Fortaleza, CE, Brazil, 2018.

	Prematurity			Low weight			1st minute Apgar ≤ 7			5th minute Apgar ≤ 7			Ventilatory support			Neonatal resuscitation		
	Yes	%	p-value	Yes	%	p-value	Yes	%	p-value	Yes	%	p-value	Yes	%	p-value	Yes	%	p-valor
**Start of PN**																		
More than 12 weeks	31	17.9	0.685*	24	13.1	0.608*	26	14.5	0.706*	3	1.7	0.740**	25	13.6	0.738*	3	1.6	1.000**
Up to 12 weeks	47	19.5		38	14.8		33	13.3		6	2.4		32	12.5		5	2	
PR/CI 95%	0.91 [0.610-1.384]			0.884 [0.550-1.420]			1.096 [0.680-1.765]			0.69 [0.176-2.744]			0.92 [0.565-1.498]			0.83 [0.202-3.449]		
**Pregnancy complications**																		
Yes	12	19.4	0.759*	8	11.9	0.610*	11	16.9	0.423*	1	1.6	1.000**	5	7.5	0.159*	0	0	0.616**
No	61	17.7		52	14.3		47	13.2		8	2.2		50	13.7		8	2.2	
PR /CI 95%	1.091 [0.625-1.905]			0.83 [0.416-1.679]			1.282 [0.703-2.338]			0.697 [0.089-5.480]			1.83 [0.760-4.433]			1.022 [1.007-1.038]		
**Number of PN visits**																		
Up to 6	39	28.3	<0.001*	33	22.6	<0.001*	18	12.8	0.701*	2	1.4	0.722**	22	15.1	0.184*	2	1.4	1.000**
7 or more	37	13.8		27	9.6		39	14.1		6	2.2		30	10.6		5	1.8	
PR /CI 95%	2.04 [1.372-3.054]			2.35 [1.474-3.755]			0.903 [0.537-1.520]			0.650 [0.133-3.180]			1.41 [0.848-2.365]			0.77 [0.152-3.934]		
**Vaccines during pregnancy**																		
No	4	33.3	0.244**	3	20	0.444**	1	7.1	0.703**	1	7.1	0.265**	1	6.7	0.705**	0	0	1.000**
Yes	70	17.8		56	13.5		57	14		8	2		54	13		8	1.9	
PR /CI 95%	1.87 [0.818-4.283]			1.48 [0.523-4.198]			0.509 [0.076-3.415]			3.62 [0.486-27.039]			1.94 [0.288-13.146]			1.02 [1.006-1.033]		
**HIV tests during pregnancy**																		
No	8	21.6	0.531*	8	20	0.252*	3	7.5	0.220*	1	2.5	0.599**	2	5	0.120*	1	2.5	0.546**
Yes	64	17.5		52	13.4		55	14.6		8	2.1		53	13.6		7	1.8	
PR /CI 95%	1.23 [0.644-2.375]			1.49 [0.764-2.915]			0.515 [0.169-1.573]			1.18 [0.152-9.205]			0.36 [0.093-1.450]			1.38 [0.175-11.008]		
**Tests for syphilis in pregnancy**																		
No	13	22.4	0.438*	8	12.9	0.790*	7	11.3	0.559*	3	4.8	0.130**	3	4.8	0.043*	2	3.2	0.317**
Yes	64	18.1		53	14.2		51	14		6	1.7		53	14.1		6	1.6	
PR /CI 95%	1.23 [0.729-2.095]			0.911 [0.455-1.821]			0.804 [0.382-1.689]			2.92 [0.752-11.400]			0.34 [0.110-1.062]			2.01 [0.416-9.765]		
**Professional who carried out the PN**																		
Both	58	18.1	0.870*	46	13.4	0.677*	46	13.7	0.577*	6	1.8	0.355*	45	13.1	0.781*	7	2	0.657#
Nurse	8	21.6		7	17.9		4	10.8		2	5.4		5	12.8		0	0	
Physician	8	18.6		7	16.3		8	18.6		1	2.3		4	9.3		1	2.3	
**Risk stratification at admission**																		
High risk	15	25	0.187*	17	26.6	0.002*	13	20.6	0.088*	2	3.2	0.627**	14	21.5	0.026*	0	0	0.611**
Normal risk	63	17.8		45	12		46	12.6		7	1.9		43	11.5		8	2.1	
PR /CI 95%	1.40 [0.859-2.298]			2.21 [1.355-3.617]			1.63 [0.940-2.851]			1.65 [0.352-7.788]			1.878 [1.092-3.232]			1.02 [1.007-1.037]		
**Delivery at maternity of residence**																		
No	42	21.5	0.185*	38	18.4	0.017*	32	15.9	0.235*	6	3	0.318**	34	16.3	0.047*	4	1.9	
Yes	36	16.4		24	10.4		27	11.9		3	1.3		23	10		4	1.7	1.000**
PR /CI 95%	1.31 [0.877-1.957]			1.76 [1.098-2.842]			1.33 [0.828-2.144]			2.22 [0.564 – 8.782]			1.642 [1.001-2.693]			1.111 [0.281-4.384]		

* Pearson Chi Square; ** Fisher Exact Test; #Likelihood ratio; PR = Prevalence Ratio; CI = Confidence Interval.

The number of prenatal consultations was associated with the outcomes: prematurity (p < 0.001) and low birth weight (p < 0.001) with prevalence ratios of 2.04 and 2.35 respectively, highlighting that these variables are directly related to adverse events in the NB.

Not having been tested for syphilis during pregnancy was associated with the outcome need for ventilatory support (p = 0.043), but with a CI that disregarded this association [0.110 – 1.062]. High-risk pregnancy stratified on admission was associated with low birth weight (p = 0.003) and ventilatory support (p = 0.026) with prevalence ratios of 2.21 and 1.87, respectively.

There was an association between fewer consultations (up to 6) and prematurity and low birth weight, increasing the prevalence of these outcomes by 2.04 and 2.35 times, respectively. High gestational risk was associated with the occurrence of low birth weight and ventilatory support, increasing their prevalence by 2.21 and 1.87 times respectively. When the delivery happened in other maternity hospital outside the municipality where the woman lived, it was associated with low birth weight and the need for ventilatory support, increasing the prevalence by 76% and 64% respectively.

## DISCUSSION

The results show the differences in obstetric care observed between health macro-regions in the same state, thus highlighting the inequalities in access to and quality of obstetric care provided in a state in the Northeast region of Brazil. In addition, the influence of the health region and the obstetric care provided on neonatal outcomes was observed. Authors state that effective prior care is directly related to a reduction in maternal and neonatal complications, favoring positive outcomes, a relationship corroborated in this study^(11)^.

The national Birth in Brazil survey found that inequalities in access to and quality of prenatal care are still evident. PN care showed significant regional differences. Despite high coverage, the proportion of pregnant women without any prenatal care was 60% higher in the North than the national average. The Southeast, South and Center-West regions had a higher prevalence of women with early initiation of prenatal care up to 12 weeks, and the Southeast had the highest coverage of women with at least six prenatal care visits^(5)^.

One region, MR Cariri, stood out in terms of a large adherence to prenatal care before 12 gestational weeks, compared with the low value of this indicator in the state capital MR, possibly due to greater coverage of care by territory or even greater difficulty in organizing the network.

An evaluation study of 689 pregnant women referred by Primary Health Care (PHC) to Specialized Care (SC) in São Paulo showed the importance of implementing the Family Health Unit (FHU) model as a strategy for health equity, as it gave pregnant women a greater chance of starting prenatal care early, as well as maintaining shared follow-up during high-risk pregnancies, as the absence of home visits increased the chance of non-sharing between services by 2.4 times^(12)^.

Access to qualified obstetric care is still a challenge, especially in regions with a disorganized healthcare network. In addition, the country’s vast territory favors the organizational inequality of health units, resulting in inadequate access and different geographical scenarios^(13)^.

Delimiting the area covered by health strategies helps to catch pregnant women early, since many women change their place of residence and/or contact telephone number without informing the health unit. In addition, there are areas that are not covered by CHWs due to a shortage of professionals, which makes it difficult to assist pregnant women^(14)^.

The low number of visits (up to 6) was associated with prematurity and low birth weight in this study. A systematic review with meta-analysis including 18 studies revealed that prenatal visits in low- and middle-income countries reduce the risk of neonatal mortality by 34% [combined effect size 0.66 (95% CI 0.54, 0.80)] compared to women who did not attend a prenatal visit. They are therefore strongly recommended during pregnancy, especially in places with limited resources, such as sub-Saharan African countries^(15)^.

Data from a prospective cohort of live births from the “Nascer no Brasil” survey, totaling 23.837 newborns, showed that inadequate prenatal care (RR = 1.71; 95%CI 1.36-2.16) was associated with neonatal near miss. This reinforces the importance of emphasizing the adequacy of prenatal care in order to identify pregnant women who need more specialized care, providing follow-up during the pregnancy-puerperium cycle to prevent undesirable perinatal outcomes^(16)^. The universal and equal access guaranteed to pregnant women can help reduce inequalities, especially for those on low incomes who need public health care services the most^(17)^.

Maternal immunization is one of the fundamental strategies aimed at achieving the third sustainable development goal, which aims to end preventable maternal and neonatal deaths^(18)^. The most discrepant prevalence occurred in the MR Sertão Central.

In a study carried out in seven public maternity hospitals and four in the supplementary health network in Belo Horizonte, Minas Gerais, an analysis of 480 pregnant women’s booklets showed that 10.63% had information on vaccination against hepatitis B; 31.46% for tetanus; and for influenza, there was no record in 90% of the booklets. There was an association between hepatitis B vaccination and paid work and the number of visits carried out in the PN^(19)^.

International studies show different explanations for the factors associated with immunization during pregnancy. A Canadian study of 1.135 health professionals found that only 56% (n = 632) reported offering vaccinations to pregnant women. The main reasons reported for not offering vaccination services in pregnancy were the belief that vaccination was outside the scope of professional practice; logistical issues surrounding access to vaccines; or a lack of staff to administer vaccines. The main factors associated with vaccinating pregnant women in practices were: professionals’ confidence in advising pregnant women about vaccines, seeing an average of less than 11 pregnant women a week and being a nurse or family doctor^(20)^.

A study carried out in the USA with data from 82.603 postpartum women showed that although almost half (48.2%) of pregnant women in rural areas had access to a funding system for prenatal care, they were less likely to live in a state that offered full coverage of care, when compared to urban areas. Among states with programs that do not offer full access for pregnant women, influenza immunization coverage was 12% lower and Tdap (tetanus, diphtheria and pertussis) immunization coverage was 20% lower for rural versus urban areas. This shows the importance of a public health system that is accessible and equitable between regions^(21)^.

Regarding the tests offered, MR Sobral had the lowest prevalence of syphilis tests among the regions, with 78.8%. A study showed that in the PN there were different barriers to early diagnosis, timely and adequate treatment of syphilis. The results of the VDRL tests on admission to the maternity hospital showed significant differences between pregnant women according to the status of their prenatal care, which hindered diagnosis and adequate treatment, favoring vertical transmission of syphilis. Among pregnant women who did not have prenatal care, there were significant percentages of infection and, consequently, high negative perinatal outcomes. And among the women who underwent prenatal care, some tested positive for syphilis, albeit to a lesser extent, missing the opportunity to control the disease^(22)^.

Another study showed that although the expansion of prenatal care coverage in the municipalities of Bahia contributed to progress in detecting cases of syphilis during pregnancy, there was no impact on reducing the incidence rate of congenital syphilis. This shows that prenatal care has limitations and needs interventions to prevent and block vertical transmission of syphilis^(23)^.

The data from the study in question is in line with the recommendations of national and international bodies when it comes to good obstetric practices based on current scientific evidence, so the professionals responsible for prenatal care are doctors and nurses in most cases. The Fortaleza MR showed a higher prevalence of prenatal care exclusively with a doctor and a lower prevalence of prenatal care exclusively with both doctors, while the Sobral MR showed a lower prevalence of prenatal care exclusively with a doctor. The literature describes that the inclusion of nurses is more often associated with the model of good obstetric practices and less intervention, thus proving the relevance of the prominent inclusion of educational practices aimed at care professionals with a reduction in interventions and, consequently, an improvement in maternal and neonatal outcomes^(24)^.

In this study, the most discrepant positive prevalences of pregnancy complications were observed in the Fortaleza and Sertão Central MRs. Data from the Birth in Brazil Survey showed that, in the SUS, 32% of women at obstetric risk were treated in hospitals without intensive care units^(25)^, which highlights the lack of structure in some maternity hospitals, making it difficult to provide comprehensive care.

High gestational risk was associated with low birth weight and the need for ventilatory support (p = 0.002 and p = 0.026). A longitudinal study based on prenatal records of 569 women in India found a prevalence of 18.3% of high-risk pregnancies, present in almost a fifth of pregnant women in rural areas, twice as common among women belonging to families below the poverty line and 41% higher in nulliparous women. Around 10.4% gave birth to low-birth-weight babies and 1.7% had stillbirths^(26)^.

High-risk pregnancies require greater structural support from maternity hospitals and prepared technical staff, given their association with unfavorable outcomes. A study of live births of 688 women followed up at the high-risk outpatient clinic of a hospital in Maringá showed that 23% of neonatal deaths were due to maternal factors. The death of newborns in the neonatal period was associated with prematurity, very low birth weight and an Apgar score of less than seven in the fifth minute of life^(27)^.

The occurrence of childbirth in the municipality of residence had a higher prevalence in the municipality of Fortaleza and a lower prevalence in the municipality of Sobral. This variable was associated with low birth weight and the need for ventilatory support. Pregnant women with diagnosed high-risk pregnancies may have been referred to larger maternity hospitals and referred to smaller municipalities, which may explain this association.

It is necessary to guarantee women’s access to childbirth care, as well as strengthening smaller municipalities to provide adequate care at this stage of a woman’s life. It is understood that, while on the one hand the referral hospital fulfills its role and attends to many women at risk during normal childbirth, on the other, it reflects the lack of quality of the small hospitals in each municipality in each region^(28)^.

As for the vitality of the newborn, as estimated by the Apgar score, there was no significant association with the PN indicators. However, a study carried out in the interior of the state of Ceará showed that the Apgar score was directly related to factors such as gestational age, maternal age group and number of prenatal consultations^(29)^.

A study of live births in Nova Friburgo, southern Brazil, showed that newborns from rural areas had a higher prevalence of extreme low weight (<1500g), low Apgar score (<5) and malformations compared to women from urban areas in the same region^(30)^.

The study reveals regional differences within the same state, showing gaps in care, even in the MR Fortaleza, which includes the state capital, as well as some health regions showing high levels of adequacy, even though they are located in the interior of the state. PN variables were associated with unfavorable neonatal outcomes, in line with national and international literature.

The study’s limitations include the fact that the data represents only one state in the country, the under-information of secondary data used in the study, and the fact that data was only collected from puerperal women, excluding other actors and variables that could help to understand the data obtained. Furthermore, it was not possible to estimate the timing of HIV and syphilis tests during pregnancy. It should be reiterated that this evaluation has provided an insight into the current obstetric context in the state of Ceará, Brazil.

## CONCLUSION

In the four macro-regions of the state of Ceará, there were regional differences in the prenatal care provided and the prenatal outcomes obtained, although good coverage of prenatal care was observed.

The association between the low number of prenatal consultations and the outcomes of low birth weight and prematurity indicates that the higher the number of consultations, the greater the possibility of timely intervention, avoiding unfavorable outcomes for the NB.

In addition, high gestational risk was associated with the occurrence of low birth weight and ventilatory support, indicating the need for structural support from maternity hospitals and technical staff prepared to stratify obstetric risk and guarantee high-complexity care, thus avoiding unfavorable neonatal outcomes. Not giving birth in the maternity hospital where the woman lived was associated with low birth weight and the need for ventilatory support, which may reflect the strategy of a care network and linking pregnant women from all over the state to the maternity hospitals of reference.

Based on the evaluation of prenatal care quality indicators and neonatal outcomes in maternity hospitals in Ceará, this research aims to provide an analysis geared towards the improvement of the care processes and positive maternal and neonatal outcomes in obstetric care, as well as encouraging managers and professionals to provide pregnant women and their children with quality care in line with good practices in pregnancy, childbirth and the puerperium. In this context, it is essential not only to draw up public policies aimed at this population, but also to implement existing policies that guarantee comprehensive care for women in the pregnancy-puerperium cycle.

## Footnotes

1 NdT: Stork Network (SN) Strategy is a term used by the MoH as a popular name derived from folk tradition of Storks delivering babies.
